# Evidence Gaps in Assessments of the Healthiness of Online Supermarkets Highlight the Need for New Monitoring Tools: a Systematic Review

**DOI:** 10.1007/s11883-022-01004-y

**Published:** 2022-02-09

**Authors:** Damian Maganja, Mia Miller, Kathy Trieu, Tailane Scapin, Adrian Cameron, Jason H. Y. Wu

**Affiliations:** 1 The George Institute for Global Health, University of New South Wales, 1 King St, Newtown, NSW 2042 Australia; 2 Global Obesity Centre, Institute for Health Transformation, Deakin University, 221 Burwood Highway, Burwood, Melbourne, VIC 3125 Australia

**Keywords:** Online supermarkets, Food retail, Food environment, Food marketing, Consumer purchasing behaviour, Digital environments

## Abstract

**Purpose of Review:**

Online grocery shopping is increasingly popular, but the extent to which these food environments encourage healthy or unhealthy purchases is unclear. This review identifies studies assessing the healthiness of real-world online supermarkets and frameworks to support future efforts.

**Recent Findings:**

A total of 18 studies were included and 17 assessed aspects of online supermarkets. Pricing and promotional strategies were commonly applied to unhealthy products, while nutrition labelling may not meet regulated requirements or support consumer decision-making. Few studies investigated the different and specific ways online supermarkets can influence consumers. One framework for comprehensively capturing the healthiness of online supermarkets was identified, particularly highlighting the various ways retailers can tailor the environment to target individuals.

**Summary:**

Comprehensive assessments of online supermarkets can identify the potential to support or undermine healthy choices and dietary patterns. Common, validated instruments to facilitate consistent analysis and comparison are needed, particularly to investigate the new opportunities the online setting offers to influence consumers.

**Supplementary Information:**

The online version contains supplementary material available at 10.1007/s11883-022-01004-y.

## Introduction

Cardiovascular disease is the leading cause of death and burden of disease across the globe [1]. Sub-optimal diet is a leading risk factor, with over one-third of all deaths from cardiovascular disease in 2019, more than 6.8 million deaths in total, attributable to dietary risks [2]. Importantly, dietary risks are preventable [3]; improving population dietary intakes can lead to rapid reductions in the risk of cardiovascular and other diet-related diseases.

Retail food environments, particularly supermarkets, are influential in shaping dietary patterns [4] in both high-income and, increasingly, in low- and middle-income countries [5]. A retail food environment that is gaining in importance is online grocery stores, accelerated by the COVID-19 pandemic. In China and South Korea, online sales were estimated to make up around a quarter of all expenditure on groceries in 2020, with considerable increases on the previous year [6]. Between April 2020 and July 2021, on average the proportion of all food retail trade in the UK (excluding Northern Ireland) from online stores was approximately double that for the year preceding [7]. In Australia, recent reporting suggests that around a third of people have started shopping online for at least some their groceries since the pandemic began [8].

These shifts in shopping behaviour make it important to understand how online retail environments are designed and how different features embedded in online supermarkets may shape consumers’ purchasing patterns.

Comprehensive and standardised frameworks and instruments to evaluate food environments are important to understand and quantify key dimensions that influence consumer behaviour [9•]. Various methodologies and tools have been developed for physical (in-store) supermarkets [4, 9•, 10, 11]. These tend to evaluate the key food environment domains defined by the 4Ps of marketing, being Product (what is available?), Price (what is cheaper?), Placement (what is seen?) and Promotion (what is highlighted or incentivised?), since these retail characteristics shape consumer preferences and choices [4, 12, 13].

While the 4Ps are likely still relevant to online stores, there is significant potential for retailers to employ new and more subtle modes of influence, for example through activity tracking and personalisation. As such, previously developed methodologies for evaluating physical supermarkets may need to be revised to capture relevant aspects of these online settings.

This review aimed to systematically search for and report on studies investigating the healthiness of real-world online supermarkets and comprehensive frameworks or collections of instruments for assessing the healthiness of online supermarkets.

## Methods

### Search Strategy

A systematic search of peer-reviewed and grey literature was conducted to identify relevant literature. This was supplemented by a manual search of the reference lists of identified reviews and other relevant papers/reports and contact with experts in the field.

The following medical/health/allied health and multidisciplinary databases were searched:

- EMBASE
- PubMed
- CINAHL
- Cochrane Library
- Scopus
- Web of Science

Search terms were developed and refined to minimise irrelevant studies and ensure relevant papers previously identified were included. Terms incorporated various aspects to effectively and comprehensively capture the concepts “food retail”, “online retail” and “food environment”.

Grey literature was searched through a review of the first 100 results on Google and Google Scholar. The following databases were also searched for relevant material:

- The World Cancer Research Fund’s NOURISHING database (https://policydatabase.wcrf.org/level_one?page=nourishing-level-one)
- The International Network for Food and Obesity/Non-communicable Diseases (NCDs) Research, Monitoring and Action Support (INFORMAS) network’s website (https://www.informas.org/)
- The Centre for Research Excellence in Food Retail Environments for Health (RE-FRESH) network’s website (https://healthyfoodretail.com/)

Search strategies are available in supplementary material (Supp. Table 1). All searches were conducted between 22 and 28 June 2021.

### Eligibility Criteria

Studies were included if they were cross-sectional descriptive studies that reported on assessments of the healthiness of real-world online supermarkets in relation to the 4Ps or other aspects of food environments that are determined by retailers (objective 1). Studies that described a comprehensive framework or set of instruments for assessing the healthiness of online supermarket environments were also included (objective 2).

Included studies were limited to real-world/natural settings to better represent implemented environments and for generalisability. Studies that assessed the healthiness of online supermarkets across settings (i.e. both supermarkets *and* other retail environments, e.g. food markets or convenience stores, both online supermarkets *and* physical stores) were included if results distinguished between settings. Studies were eligible if they were published after 1 January 2010 to ensure relevance to contemporary environments and features in this rapidly developing context. Original studies and reviews were eligible for inclusion and studies must have been available in English, Spanish, Portuguese, Italian or German. Reasons for exclusion were documented.

### Screening and Selection of Articles

Duplicates were removed prior to screening. The titles and abstracts of references retrieved from searches were screened by a review author (DM) to identify papers/reports that potentially met the eligibility criteria. Full-text copies of potentially relevant sources were then retrieved and independently assessed by two review authors (DM, MM) to identify those that met the inclusion/exclusion criteria. Discrepancies were resolved by discussion and consensus. A data extraction form, developed to collate data on study setting, design, findings and food environment domain assessed, was populated by one review author (DM).

## Results

### Study Selection

A total of 2519 references were retrieved from searches of peer-reviewed literature, with a further 86 records identified through grey literature and other sources. After removing duplicates, 1286 records underwent title and abstract screening. Of these, 203 full-text items were sought for further assessment against inclusion criteria. Finally, 18 studies were eligible for inclusion: 17 for objective 1 and one for objective 2. A flow-diagram of the search and selection process is included in supplementary material (Supp Fig. 1).

### Studies Investigating the Healthiness of Online Supermarkets (Objective 1)

Seventeen papers detailing assessments of the healthiness of real-world online supermarkets were included in our analysis. Organised by the 4Ps, noting that some studies investigated multiple elements, 11 reported on prices, nine on products (predominantly availability of nutrition information) and five on promotions, with no papers reporting on placement. Twelve analysed data from the past 5 years (since 2016), with one paper not specifying the period of data collection. Almost all were conducted in high-income countries (*n* = 15, 88%; six in the UK, five in Australia, one each in New Zealand, Germany, Ireland and the USA), with an additional two (12%) studies in an upper-middle-income country (Brazil). No studies in lower-middle- or low-income countries were identified. Relevant characteristics of studies included under objective 1 are presented in Table 1.

**Table 1:** Relevant characteristics of included studies (objective 1)

Study and location	Setting	Design	Brief overview of main relevant findings	Food environment domain/s assessed
Bhatnagar et al. 2021 [14], UK	Six physical supermarkets, time period unspecified; seven online supermarkets, exact time period unspecified but data collection started in November 2017	Data collected from randomly sampled products in physical stores, with those products matched to product data collected from online stores. Product availability, prices, price promotions, reported nutrition information and display of front-of-pack labels compared between physical and online settings	Product availability, prices and reported nutritional values were similar between settings. Online products were less likely to display front-of-pack nutrition labelling (42% online vs 74% for physical stores) and have price promotions applied (24% online vs 32% in-store)	Product, price
Cameron et al. 2017 [15], Australia	Four online supermarkets, June–September 2013	Weekly online catalogues captured to assess healthiness of included products. Products categorised by food group, then as “core” (recommended for a healthy diet), “discretionary” (should consume rarely and in small quantities), alcohol or other foods, as per relevant Australian government guidance. Number of products and catalogue pages dedicated to products described and compared between retailers	Discretionary products were more likely to be included in catalogues than core products overall (43.3% discretionary vs 34.2% core) and across all retailers. Alcohol was less likely to be included than core products overall (8.5%) and for all retailers, while other foods made up the remaining 14.0%	Promotion
Furey et al. 2019 [16], Ireland	Two online supermarkets, March 2016–February 2017	Information on products displayed on “top offers” section of website collected to assess healthiness of price promotions. Data included product attributes, prices, types and magnitude of price promotions and nutrition information. Products were categorised according to food type (as per relevant government guidance), with the category “foods and drinks high in fat, sugar and salt” denoting unhealthy products, and healthiness (nutritional quality score, based on traffic light labelling thresholds)	Price discounts were the most common form of price promotion (40.6%), followed by offers listed without reference to pre-promotional price or discount (37.0%) and multibuys (17.6%). Overall, foods and drinks high in fat, salt and sugar were the most commonly promoted category (34.4% of total promotions). These products constituted a greater proportion of total promotions in winter (42.0%) and a lower proportion in summer (26.6%). Price promotions overall were least healthy in spring (nutritional quality score 1.99, 35.7% of products with red traffic light) and most healthy in summer (nutritional quality score 2.14, 26.4% of products with red traffic light)	Price
Goulding et al. 2020 [17], Australia	One online supermarket, August 2019	Two shopping baskets, a “planetary health diet” and a “typical diet”, were developed to assess affordability of a healthy and sustainable diet. Prices were captured using delivery postcodes representing the lowest, middle and highest quintiles of socioeconomic advantage and disadvantage in each capital city. Prices for each postcode were compared	The more healthy and sustainable basket of products was cheaper than the reference diet and more affordable overall (median cost $188.21 vs $224.36 per basket, median affordability 13% vs 16%) and in every city and socioeconomic group	Price
Harrington et al. 2019 [18], UK	Six online supermarkets, March 2018	Automated weekly data collection from online supermarkets captured number, prices and nutrient content of products and additional information available on product pages such as ingredients lists. Data on number, prices and information at a single time-point was reported. Nutrient content, prices, eligibility to display traffic light front-of-pack labels and “healthiness scores” (determined by traffic light labels) of ready meals and pizzas were reported	Over three-quarters of individual product pages provided information on ingredients and on nutrition information overall (80.9% and 85.9%, respectively) and in each store. Over 90% of products overall reported energy, protein, carbohydrate, fat, sugar, salt and saturated fat content, while fibre was declared on over two-thirds of products. A majority of ready meals and pizzas were eligible to display green traffic lights for sugar. Cheaper ready meals had lower fat, saturated fat, sugar and salt content and better healthiness scores. Cheaper pizzas reported lower salt content	Product, price
Hillen 2021 [19], Germany	One online supermarket, May 2019–March 2020	Prices for products in 13 food categories collected near-daily over data collection period, with prevalence of psychological pricing (where a price is set slightly lower than a round number, typically ending in 9, to create a perception that a product is meaningfully lower in price and thus encourage purchase) by type of pricing (normal or discounted) and food category assessed	Over two-thirds of prices ended in 9. Products on sale and more expensive products overall were less likely to display prices ending in 9. Psychological pricing was most commonly applied to frozen products and sweets and snacks, and least likely to be applied to fruits and vegetables	Price
Obesity Action Scotland 2021 [20•], UK	Six online supermarkets, March and November–December 2020	Two shopping lists (healthy and standard) were created based on previous studies. Products were categorised as “discretionary” (high in fat, sugar and salt), as per relevant government guidance. Online accounts were created for each of the supermarkets, browser history and data were cleared and a standard protocol used to search for the two baskets. Promotions were categorised as price (e.g. discounts) or non-monetary (e.g. items being featured or displayed prominently). Prevalence and healthiness of price promotions were assessed	Each shopping event displayed, on average, 510 promotions. Non-monetary promotions (61%, of which 46% were products displayed when adding products to cart, including after searches, and 37% promotions on a separate offers page) were more common than price promotions (39%, of which temporary discounts comprised 57% and multibuys 39%). Twenty-one percent of promotions were for unhealthy products (most commonly crisps and confectionery), with an additional 11% of promotions for alcohol; the remaining 68% of promotions were on products not classified as discretionary. For each supermarket, the types of products promoted differed according to which basket of items was selected. On average, more items overall and a greater number of unhealthy products and alcohol were promoted in November/December than in March	Price, promotion
Ogundijo et al. 2021 [21], UK	Three online supermarkets, time period unspecified	Data on 500 randomly selected packaged products, across five categories and three supermarkets, collected from product pages to assess healthiness of products and display of nutrition information. Relevant information captured included labelling format and nutrition, allergen and ingredient declarations. Types of nutrition labelling on products were identified. Products were also categorised, based on reported nutrition content, against the UK traffic light labelling criteria	69.8% of products carried a front-of-pack label, with traffic light labelling and guideline daily amount labelling most common; 48.0% displayed interpretive labelling (traffic lights). 27.7% of products displayed a nutrition information panel. 5.2% did not display any nutrition information. Around half of products would be categorised as medium or high in total fats (51.2%), total sugars (48.0%) and saturated fats (45.2%), and almost a third would receive a medium or high in salt (31.0%)	Product
Olzenak et al. 2020 [22•], USA	Twelve online supermarkets, August–September 2018	A list of 26 products across major food groups was selected, with a standard protocol for identification of products used for each of the online stores. Information on search functionality and availability of nutrition information and/or ingredient declaration was recorded. Location of nutrition information (immediately visible, visible with scrolling, visible with a click away from initial page) was captured and legibility was further assessed through set criteria. Study aimed to identify availability of nutrition information and store navigation features	Eighty-two percent of individual product pages displayed nutrition information and 82% displayed ingredient information. Products which are required to display this information in physical settings by regulation are more likely to provide it than those which are not required to display this information, e.g. fresh produce (for nutrition information 85% vs 46%, for ingredients information 85% vs 54%). Nutrition information was most commonly located away from the initial product page (53.3% of total displaying nutrition information). Legibility of nutrition information varied across product categories (range 9 to 100%). Most stores offered filtering of search results by at least one nutrition-related attribute, most commonly for gluten-free products (75%), and none provided an option to sort by a nutrition attribute	Product
Pearson et al. 2014 [23], New Zealand	One online supermarket, 16 physical supermarkets and 8 food markets, March 2013	Eight common, often locally grown and relatively low-priced fruits and vegetables were selected for purchase from a range of outlets, with prices recorded. Products were compared between settings, both individually and as a combined basket	Some fruits and vegetables were cheaper online than in physical supermarkets, while for others the opposite relationship was found. The basket of fruit and vegetables was cheaper online ($113) than in physical supermarkets ($124, $127) and farmers’ markets ($138), but more expensive than other fresh food markets ($76)	Price
Pereira et al. 2019 [24], Brazil	One online supermarket, May–July 2017	Products in categories not exempt from regulations on nutrition labelling were collected for the assessment of the prevalence of health and nutrition claims. A randomly sampled subset of these products was further examined for price, nutrition labels and marketing techniques applied, declared ingredients and nutrient content. Nutrition/health claims and marketing techniques applied were categorised, with ingredients lists used to classify products against the NOVA food processing classification system. Three different price measures were calculated	A majority of products (60.6%) were categorised as ultra-processed. 32.8% of products displayed health or nutrition claims, predominantly nutrition claims (displayed on 31.3% of total products), with claims of the benefits of certain nutrients most common (74.9% of total claims). Dairy and packaged cereal products were most likely to display claims. 49.0% of products utilised some form of marketing technique, predominantly promotion of health and well-being and promotion of naturalness (29.6% and 29.1%, respectively, of marketing techniques applied). A majority of products displaying claims and utilising promotional techniques were ultra-processed foods (70.0% and 64.0%, respectively). 66.1% of promotions of health/wellbeing and 56.4% of promotions of naturalness were on ultra-processed products. Products displaying at least one claim had lower levels of total fats, saturated fats, trans fats and fibre, while products applying marketing had less total fats, saturated fats and fibre. Processed and ultra-processed products overall had higher energy, total fats, saturated fats, trans fats and sodium, while total carbohydrates, protein and fibre were higher in unprocessed products. Processed and ultra-processed products were more expensive, per 100 g/mL and per 100 kcal energy, than unprocessed products	Product, price, promotion
Pereira et al. 2021 [25], Brazil	One online supermarket, May–July 2017	A random set of products in categories not exempt from regulations on nutrition labelling was collected. Product attributes, nutrition/health claims, marketing techniques, ingredients lists and reported nutrient content were captured. Products were classified against the NOVA food processing classification system. Four different nutrient profiling models were applied to products, with proportions passing/failing each model calculated and compared. The four models are ordinarily used for diverse purposes, i.e. to assess eligibility to display health/nutrient claims, assess eligibility to be marketed to children, identify products unsuitable for marketing and identify products for reformulation	A majority of products were categorised as ultra-processed (60.6%). There were considerable differences in the proportion of products passing each of the four nutrient profiling models and the food categories included. For all models, ultra-processed products were least likely to pass (range 40.4 to 15.8% of products passing) and unprocessed products were most likely to pass (range 93.1 to 79.3% of products passing), but ultra-processed products were still the most common products passing in three of the models (range 42.8 to 29.9% of total products passing). For three of the four models, products displaying health/nutrient claims were more likely to pass. The use of marketing techniques was not associated with pass rates. Nutrient composition in products passing differed by model, but sodium was lower in passing products for all four models and saturated fats and added sugars were lower for passing products in three of the models	Product, promotion
Price et al. 2017 [26], UK	Four online supermarkets, April 2014–April 2015	Nutrition information was collected from products promoted in special offers sections of online stores. Healthiness was assessed by “nutrient quality” scores (based on thresholds included in traffic light labelling) and food types, classified according to relevant government guidance. The category “high fat high sugar foods” denoted unhealthy products. The prevalence of promotions was also compared against the proportion of a diet that should be dedicated to particular categories, again according to relevant government guidance	Promotions were most commonly found on products with “medium” nutritional quality (37% of promotions), while promotions were least likely on “low” quality products (29% of promotions). Products high in fat and sugar were most commonly promoted (33% of promotions, compared to recommended 7% of intake), while fruits and vegetables (14% of promotions, compared to 33% of recommended intake) were most under-represented in promotions	Promotion
Riesenberg et al. 2019 [27], Australia	One online supermarket, April 2017–April 2018	Product data from 11 categories was collected, with product attributes, price, promotional price and type of promotion captured to assess prevalence and magnitude of price promotions. Products were classified as “core” (recommended for a healthy diet) or “discretionary” (should consume rarely and in small quantities) according to relevant government guidance. A summary indicator of overall healthiness, the Health Star Rating (HSR, where higher represents a more healthy product, on balance), was calculated for products. Seasonal variation was also captured	Discretionary products were almost twice as likely to be discounted (28.8% vs 15.1%), saw greater relative discounts (− 25.9% vs − 15.4%) and were more likely to be discounted through multibuy promotions than core products (3.6% vs 1.9%). Similarly, products with a lower HSR were more likely to be discounted than products with higher HSRs (32.4% of promoted products had an HSR of 0.5 and 15.9% of promoted products had an HSR of 5) and saw bigger discounts. Ice cream and frozen fruit were more likely to be discounted in summer than in winter, while confectionery was more likely to be discounted in winter than in summer	Price
Stones 2016 [28], UK	Five supermarkets with online and physical stores, July 2015	Twenty products (10 branded and consistent across supermarkets, 10 private label), across a range of common product categories, were identified per supermarket. A three-part categorisation system was used to assess the display of nutrition information. Extent of nutrition labelling was compared within and between online supermarkets and between online and physical settings	No online stores displayed nutrition labelling on search screens. On product pages, the most commonly displayed nutrition labelling overall was a combination of a traffic light summary, black and white nutrition information panel and recommended intake label (31% of total products). Traffic light summaries appeared on less than half of products (approx. 41%). Only one product (1%) did not display a nutrition information panel. Across the supermarkets, the prevalence of each labelling type/combination differed considerably. Seventy-three percent of products, and a majority of retailers, only displayed nutrition information upon scrolling down a product page. Online products generally featured fewer and/or less accessible nutrition labels	Product
Zorbas et al. 2019 [29], Australia	Two online supermarkets, November 2016–November 2017	Data was collected for non-alcoholic beverages weekly, in line with price promotion cycles, to assess prevalence and magnitude of price promotions. Product attributes, price, promotional price and type of promotion were captured. Products were classified as sugar-sweetened, artificially sweetened, 100% juice and flavoured milk, and plain milk and water. The proportion of products price-promoted was also tracked across the time period	Sugar-sweetened beverages (excluding flavoured milk and 100% fruit and vegetable juice) were the most common beverages (40.1% of total). All sugary drinks (including flavoured milk and 100% fruit and vegetable juice) constituted 66.1% of total beverages available, with an additional 13.5% artificially sweetened beverages. Sugar-sweetened beverages were the most commonly price-promoted beverages, comprising almost half of all beverage promotions (47.8% of total price promotions). Artificially sweetened beverages were most likely to be discounted. Plain milks and waters had the fewest promotions and were least likely to be promoted. Sugar-sweetened beverages and artificially sweetened beverages were most heavily discounted. A majority of multi-buy promotions were for sugar-sweetened beverages	Product, price
Zorbas et al. 2021 [30], Australia	Sixteen physical supermarkets and two online supermarkets, October 2018	Product availability and price were compared between the physical and online supermarkets. Products were categorised by food type and as “discretionary” (consume rarely and in small quantities) as per relevant government guidance. Using online data, price and affordability of two reference diets (healthy and discretionary) and the prevalence of price promotions were also assessed. Two separate measures of affordability were calculated	There was near perfect agreement between online and physical settings on product availability, price and price promotions overall and by product healthiness. A healthy diet was calculated as 21% cheaper than an unhealthy diet. The healthy diet was assessed as affordable under one threshold and unaffordable under another. Price promotions were infrequent (applied to 11% of items on average) and did not appreciably improve affordability	Product, price

#### Pricing

Several studies compared the costs of hypothetical diets and actual products. Two Australian studies found that a healthy diet was cheaper than a reference “typical” diet [17, 30], while others identified that healthier ready meals and unprocessed products were cheaper than their less healthy counterparts [18, 24].

A New Zealand study found that a basket of fruits and vegetables was cheaper from an online supermarket than physical supermarkets [23]. Two studies investigating the prevalence of price promotions on online supermarkets and consistency in pricing and price promotions between online and physical stores found that prices were similar across settings [14, 30]. However, in the Australian study, price promotions were infrequent overall (applied to 11% of products) and consistent across settings [30], while in the UK study, price promotions were more common but online stores were less likely to display them (24% online vs 32% in-store) [14]. Analysis of data from six online supermarkets in Scotland found that price promotions were less frequent than non-monetary promotions [20•].

Three studies found that unhealthy products were more likely to be price promoted than healthy foods. In Ireland, products high in fat, sugar and salt (as a category) were most commonly price promoted [16]. In Australia, one study found that unhealthy products were almost twice as likely to be discounted [27], with another finding that almost half of total beverage price promotions were for sugar-sweetened beverages [29]. Both also found that unhealthier products were most heavily discounted [27, 29]. Seasonal variation in the unhealthiness of price promoted products was also identified [16, 27]. An analysis of data from an online retailer in Germany identified that “psychological pricing”, where a price is set slightly lower than a round number (typically 9) to create a perception that a product is meaningfully lower in price and thus encourage purchase, was both common and most commonly applied to less healthy foods [19].

With regard to the types of price promotions, a study in Ireland and a study in Scotland both found that discounting was more common than multibuy promotions (where consumers are incentivised to buy additional products) overall [16, 20•]. However, two Australian studies found that multibuys were more commonly applied to unhealthy products [27, 29].

#### Products

The few available studies suggest that a large number of unhealthy products are typically provided in online supermarkets. In one UK study, around half of all products with declared nutrient content would be categorised as medium or high in total fats, total sugars and saturated fats, and almost a third would be categorised as medium or high in salt [21]. An Australian study identified that around two-thirds of total beverages available were sugary drinks and only one in five were plain waters or milks [29]. Two studies in Brazil found that three-fifths of products not exempt from regulations on nutrition labelling were classified as ultra-processed and a further fifth as processed [24, 25]; processed and ultra-processed products were less healthy on average [24] and ultra-processed products were the most common products passing in three of four nutrient profiling models used to target/enforce various food policies [25].

Several studies assessed the availability of nutrition information. Results differed widely, even within countries and across similar time periods. An analysis of three UK online supermarkets (period of data collection unknown but paper published in 2021) found that while two-thirds (69.8%) of products displayed some sort of front-of-pack nutrition label, just under half (48.0%) provided interpretive labelling (traffic light labelling), around a quarter (27.7%) displayed a nutrition information panel and 5.2% did not provide any nutrition information at all [21]. Another study of five UK online retailers (data collected July 2015) found that only one product (1%) did not provide the required nutrition information panel and that interpretive traffic light labelling was only present on approximately 41% of products [28]. A third UK-based study of six online supermarkets in March 2018 found that over three-quarters of product pages provided nutrition information (85.9%), with a similar proportion (80.9%) also providing information on ingredients [18]. Another study, analysing data from the same database (exact period of data collection unknown, but collection for the database commenced November 2017), reported that only 42% of products provided front-of-pack nutrition labelling of any type [14]. Two of these studies also compared products available online and in physical stores, identifying that products available online were less likely to provide front-of-pack nutrition labelling [14] and that online products generally featured fewer and/or less accessible nutrition labels [28].

Outside of the UK, a study in the USA reported that while a large majority of product pages do display nutrition and/or ingredient information, not all products which must provide nutrition information by regulation do so [22•].

While nutrition information may be available, the location of this information is also important and has been considered by two studies. In one UK study, nearly three-quarters of products only displayed nutrition information upon scrolling down a product page, while those retailers who did display nutrition information at first glance did not do so across all their products [28]. The USA study also found that information was mostly not presented immediately, with over half of products for which nutrition information was available presenting this on a different page [22•].

The two previously mentioned Brazilian studies also looked at health and nutrition claims. Almost a third of products displayed claims, with over two-thirds of products displaying claims ultra-processed, though products displaying claims were generally healthier than those not displaying claims [24]. Products displaying health/nutrient claims were more likely to pass three of the four nutrient profiling models applied to these products [25].

One study in the USA looked at the ability for users to customise online stores to suit their nutrition needs, identifying that products could be filtered by attributes such as fats or sugars content in half or fewer stores, respectively, and no stores offered the ability to order product listings by nutrition content [22•].

Two studies that compared products in online and physical stores, one in the UK [14] and one in Australia [30], found that product availability generally aligned across the settings.

#### Promotions

Only one study reported on the prevalence of promotions on online stores. A study in Scotland found that shoppers were exposed to over 500 promotions on each shopping occasion, on average, with non-monetary promotions more common than price promotions [20•]. Forty-six percent of all non-monetary promotions were products displayed when selecting products (on product pages and search results), with a further 37% found on a separate offers page.

Findings on the healthiness of promoted products differed. An Australian study found that unhealthy products were most commonly advertised [15] and a study from Northern Ireland found that products high in fat and sugar (as a category) formed the greatest proportion of promotions overall [26]. In this latter study, unhealthy products were most over-represented (33% of total promotions, compared to recommended 7% of intake), while fruits and vegetables were most under-represented (14% of promotions, compared to 33% of recommended intake) [26].

In the study of six online supermarkets in Scotland, only a fifth (21%) of promotions were for unhealthy products, most commonly crisps and confectionery, with an additional one-tenth (11%) of promotions for alcohol [20•]. As evidence of the sophisticated capacity of online retailers to tailor the displayed environment to users, the types of products being promoted differed in real-time according to whether a healthy or a standard basket of items was being selected. This study also found seasonal variation in the healthiness of promotions.

One Brazilian study, assessing the prevalence and messages of marketing techniques on products, found that almost half utilised some form of marketing [24]. 64.0% of total marketing techniques applied, including 66.1% of promotions of health/wellbeing and 56.4% of promotions of naturalness, were on ultra-processed products. Overall, however, products using promotional techniques had less total fats and saturated fats and more fibre.

### Frameworks and Instruments for Assessing the Healthiness of Online Supermarkets (Objective 2)

While each of the studies included under objective 1 necessarily involves the use of some form of instrument for capturing and assessing an aspect of the retail food environment, none offered a comprehensive or consistent set that can be systematically applied in other studies. In addition, none sought to outline or describe the entire online supermarket environment, as the first step in developing a coherent set of readily applicable tools.

One additional paper providing a conceptual framework to comprehensively capture the healthiness of online retail food environments was identified (Khandpur et al. 2020 [31••]). The framework was developed through key informant interviews, literature reviews and pilot testing. An expert group, consisting of researchers and technical experts in online food retail, also participated in trial shopping exercises and discussions with authors to further refine the framework and assess content validity.

This framework aims to capture the entirety of the online retail environment’s influence on consumer behaviour. It incorporates attributes that are both common to physical settings, at least at a higher level, and specific to the online environment.

Domains outlined under this setting are classified as “dynamic”, signifying aspects of the environment that are determined to some extent by consumer-online system interaction, i.e. potentially modified by the retailer or the consumer themselves, or “static”, indicating attributes that are likely consistent for all users. The domains included are retailer policies and practices pre-shop (e.g. inventory management, website access) and post-shop (e.g. delivery, cancellations and orders) and consumer characteristics, preferences and past behaviours (static); and personalised marketing by retailers, marketing outside of the online store setting and consumer customisation of the website (dynamic). The framework also provides “cross-cutting” domains to capture equity and transparency in retailer policies and practices and the social, community and policy context in which online shopping develops and occurs.

The 4Ps are referenced under the domain “personalized marketing by retailers”, though the constructs outline different manifestations of the 4Ps in an online supermarket. This domain encompasses those mechanisms through which retailers adapt the online store environment to individual consumers, i.e. the immediate determinants of how the online supermarket environment looks and feels that can be manipulated by retailers to influence choices. Table 2 provides an overview of the elements included under this domain, as these were the focus of this review, as well as papers identified through this review that consider aspects of those attributes. Note that the studies are mapped according to the guidance provided by Khandpur et al., e.g. products promoted on separate special offers pages are included under “time-limited deals”, nutrition labelling is included under “point-of-purchase information” and products suggested after an earlier purchase under “cross-promotions” or “recommendations”.

**Table 2:** Online store elements and description under the domain “personalized marketing by retailers”, from Khandpur et al. Table 1 [31••], mapped to identified studies

Personalized marketing by retailers		Identified studies
Construct	Description	
Product — product mix	These include the variety, brands and assortment of products the consumer can view on the online platform	[14, 18, 21, 24, 25, 27, 29, 30]
Price — discounts	Examples include lower prices on targeted products (discounts, two-for-one deals, cost-saving strategies) which may be open to all customers or exclusive to members of loyalty programs	[14, 16, 19, 20•, 27, 29, 30]
Price — rewards	Rewards include links to coupons, loyalty programs, membership rewards and other redeemable rewards	[20•]
Price — time-limited deals	These include special deals that are valid for a set period (24 h, 3 h, etc.) or weekly flyers meant to incentivize food purchase within a specific period of time	[15, 16, 20•, 26]
Placement — cross-promotions	Examples include marketing of complementary products anchored to a previous search or to items already in the shopping cart (milk and eggs suggested on a search results page for bread or milk suggested at checkout when cereal is in the shopping cart)	[20•]
Placement — search result order	Examples include non-random presentation of products (search results ordered by the most expensive products or display of sponsored products before other items)	No studies identified
Placement — recommendations	Examples include seasonal products, popular items, recently viewed products, suggestions based on past purchases, recommended product/brand swaps or impulse buys (cookies or candy recommended at checkout)	[20•]
Promotions — advertisements	These include products on paid banner advertisements or title cards (large panel of images or text at the top of a page) displayed on the website that link to a separate landing page featuring the sponsored product	No studies identified
Promotions — branded site content	Examples include branded products integrated into the existing site content, like department images (branded cereal displayed to indicate the breakfast cereal department), branded recipes or meal solutions (branded marinara sauce depicted in a lasagna recipe), promoted product swaps and retailer-generated shopping lists	No studies identified
Promotions — user feedback	This includes highlighting consumer product reviews and ratings to promote the selection of certain products	No studies identified
Promotions — social media	Examples include links to the retailer’s Instagram, Facebook or other social media pages promoting specific brands or products and opportunities for consumers to share purchased products through personal social media accounts	No studies identified
Promotions — point-of-purchase information	These include labels, nutrient and health claims (non-GMO, whole-grain) and other product descriptors (product source, organic) that may be personalized to promote the selection of certain products	[14, 18, 21, 22•, 24, 25, 28]

Findings from our review also suggest that non-discounted prices (i.e. standard pricing in the absence of discounting or promotions), not clearly defined or captured through the above, may also be important [14, 17–19, 23, 24, 30]. Online store layout and navigation beyond the facets discussed above (e.g. placement on front, category and sub-category pages) may be another attribute worth investigating [20•, 32, 33].

Khandpur et al. did not provide, alongside the framework, a ready-made instrument that could be applied to consistently audit the healthiness of online retail environments. No results of application of the final framework against real-world supermarkets have been reported as yet.

## Discussion

Practices applied in retail environments strongly influence purchasing and dietary patterns [4], and therefore have the potential to impact cardiovascular health [34, 35]. Our systematic review has highlighted that a small but growing number of studies are assessing the healthiness of certain aspects of online supermarkets, but overall the evidence remains limited. In addition, until recently, no systematic, overarching conceptual framework, identifying relevant practices that should be investigated further, has been available to guide consistent approaches to such assessments.

Despite the limited evidence, a number of interesting patterns have emerged based on the studies identified here. Price was the most regularly assessed online retail domain. However, while analysis of prices can inform assessments of the affordability of healthy and unhealthy products/diets, this is unlikely to be sufficient to suggest whether an online supermarket actually encourages their consumption. Some studies in the online setting have identified that a healthier diet may be cheaper than unhealthy diets/products [17, 23, 24, 30], but online supermarkets may undermine such intentions through misleading or deceptive pricing strategies [19], as commonly seen in physical supermarkets [36].

In relation to product placement and promotions, the advanced capability to disguise healthier products in this setting raises concerns. A number of studies identified through this review focussed on price and other promotions and advertising, which is an important avenue through which purchases and preferences are influenced. These suggest that promotions are largely skewed towards unhealthy products [15, 16, 20•, 24, 26, 27, 29]. This phenomenon is not unique to the online setting, however, with a recent review also finding that they were more frequently applied to less healthy products in physical supermarkets [37].

In addition, the focus on food labelling (basic nutrition information, interpretive nutrition labelling, ingredient and allergen information and health and nutrition claims) identified through this review [14, 18, 21, 22•, 24, 25, 28] is warranted, particularly as interpretive nutrition labelling has been shown to improve dietary choices [38, 39] but may be less common online than in physical stores [14, 28]. Our review suggests that the display of information online frequently does not meet mandated requirements for the same products in physical stores, or appear frequently enough to effectively support consumer decision-making, indicating that greater attention may need to be paid to updating regulation to account for these environments and/or monitoring compliance. The potential application of such labelling for marketing purposes, above encouraging informed and healthier choices, should also be closely monitored. Again, these issues are not restricted to online supermarkets; limited uptake of interpretive nutrition information on product packaging [40] and the use of health and nutrition claims on unhealthy products [41–44] is seen in physical supermarkets in countries where such labels may not be effectively regulated.

It is critical to comprehensively and, given their capacity to rapidly evolve, regularly investigate these environments. The studies reported here suggest ways to adapt existing and create new tools and protocols to examine these environments more closely; however, a coherent set of validated instruments to approach the task systematically and enable consistent analysis and comparison is needed.

The guiding framework described by Khandpur et al. is therefore an important and timely addition to the literature [31••]. Some of the elements in the framework align with the myriad auditing/assessment tools developed for use in physical retail environments [4, 9•, 10, 45–48]. This is clearly seen in the studies identified through this review, which largely applied or adapted existing methods for evaluating the 4Ps in physical stores.

However, the additional opportunities that the online environment introduces for retailers to manipulate consumer preferences and choices have been less well studied. The clear gaps in the literature, as shown in Table 2, may therefore also be due to a lack of frameworks and tools to capture the *different* ways that online supermarkets can influence choices. Though some papers identified here do consider some of these (placement of nutrition labelling [22•, 28], placement of promotions [20•], online store layout/navigation [20•], potential for user customisation [22•, 28]), studies to date have largely not uncovered any information specific to the online environment. This highlights the need for considerable methodological development.

Unfortunately, there have been only few investigations into the new ways that online supermarkets might influence consumer purchases and dietary patterns, particularly in the medical and health literature. One notable study tracking the information used by consumers shopping for groceries online found that site navigation was the most common method to identify relevant products, followed by searches and then pages displaying special offers [33]. Findings also suggest the importance of product photos and a lack of attention given to details such as nutrition information and other indicators of healthiness (traffic light labels, in this instance).

Another study, analysing data on nearly 200 million online transactions from a single UK retailer, provides some insight into aspects largely unique to the online setting [49]. Elements investigated included price sensitivity (selection of products from offers, deals and flash sales and after sorting products by price in ascending order) and “basket stability” (adding a product from a shopping list, favourites, suggested orders and previous orders) or “disrupted” activities (adding a product from searches or after engagement with offers and other features). Overall, few products in a shopping basket were added through price sensitive behaviours; of those products selected via a price sensitive mechanism, the vast majority were from special offers, which are more likely to be displayed prominently on the website. A majority of product selections were due to disrupted activities, predominantly searches.

A third useful study investigated the influence of “in-store displays” in an online supermarket, categorised as first screen, “aisle” (brand-level) and “shelf” (product-level) [32]. The authors found that these displays increased sales overall, with first screen displays more effective than aisle displays, which were more effective than shelf displays, i.e. products displayed before alternatives were available were more effectively promoted.

There is also some evidence to suggest that consumers’ use of mobile technologies (e.g. phone or tablet) for online shopping is associated with increased number of orders, and that consumers using mobile technologies are more likely to purchase habitual products [50].

Though automated online data collection methods are increasingly used and can be used to effectively and efficiently build a large dataset, they also have their limitations. While the routine collection of data from online supermarkets may be useful to describe some aspects that influence purchases and/or the healthiness of purchases (e.g. product nutrition information, price), or to investigate longitudinal changes in the same [18, 30], they may not capture all relevant information such as temporary promotions and product placement. Furthermore, automated methods largely capture elements that apply equally to online and physical stores, not those different features in the online setting that introduce new methods to influence consumers. In addition, it is evident that assessments of the overall online retail environment within a country should attempt to include as many retailers, within similar timeframes, as possible. Inconsistencies in such study design features likely underpin the wildly diverging findings on proportions of products displaying nutrition information observed across studies included in our review [14, 18, 21, 28].

Key strengths of our study include a comprehensive search for peer-reviewed and grey literature across a number of sources, including outside the medical and health research spheres. It also focussed on real-world settings in an attempt to understand these retail food environments as they actually exist and to improve generalisability.

However, there are some limitations to our review. All included studies but two were conducted in a small number of high-income countries, limiting the generalisability of our findings. Other avenues for consumers to purchase groceries online, for instance through convenience- or corner-type stores and via online food delivery services, are also likely to become increasingly common but were not considered here. In addition, the focus on real supermarkets has excluded the considerable number of studies, both observational and interventional, that have used simulated environments to understand elements of online supermarkets and strategies that may influence consumer choices.

## Conclusions

The impact that the shift towards online grocery shopping will have on purchases, diets and health is currently unclear. However, our systematic review suggests that online supermarkets are already skewed towards promoting unhealthy products and, drawing on findings from the greater number of audits of physical stores, retailers are clearly willing to apply various strategies to encourage unhealthy choices. We have also identified that nutrition labelling in online retail environments is likely not sufficient to support informed consumer decision-making.

The online environment offers new and more covert methods to further the bias towards the promotion of unhealthy ultra-processed foods. As such, these environments must be closely monitored for the potential to direct consumers towards unhealthy products and diets, including a focus on identifying practices affecting or targeting vulnerable groups as a specific priority. There is also a need for further work to empirically identify and understand how consumer choices, and thus dietary patterns, nutrient intakes and health, are impacted by online retailing practices.

Paying heed to identified issues in measuring and comparing the healthiness and impact of food environments [9•], a comprehensive framework, such as that developed by Khandpur et al. [31••], provides a crucial reference point for future assessments of online supermarkets. Many of the studies identified in this review provide some insight into relevant aspects of that framework. However, more must be done in terms of developing validated and coherent tools to audit contemporary online supermarkets and consistently applying them to understand the various retailing and marketing practices involved. Studies which investigate the extent to which real-world online supermarkets preferentially apply these strategies to unhealthy products are then required to recognise, and act to improve, those settings which encourage unhealthy dietary patterns and increase the risk of diet-related disease.

The availability of a standardised but readily adaptable protocol (for example through the INFORMAS retail module, which aims to develop and apply common methods to monitor food environments across the world [51]) will be critical. This will support assessments in broader settings than those identified in this review, as online stores will continue to expand within and outside of high-income countries. Consistency in approaches will also allow comparisons between retailers and over time. Such efforts are essential to inform policy making, both by government and retailers, that supports healthier online supermarkets, improved dietary patterns and reduced risks of diet-related disease.

## Supplementary Information

Below is the link to the electronic supplementary material.

Supplementary file1 (DOCX 50 kb)

## References

[1] Roth GA (2020). Global burden of cardiovascular diseases and risk factors, 1990–2019: update from the GBD 2019 study. J Am Coll Cardiol.

[2] Institute for Health Metrics and Evaluation, GBD compare. 2021, IHME, University of Washington: Seattle, WA.

[3] GBD 2017 Diet Collaborators, Health effects of dietary risks in 195 countries, 1990–2017: a systematic analysis for the Global Burden of Disease Study 2017*.* Lancet, 2019;393(10184):1958–1972. 10.1016/S0140-6736(19)30041-8 PMC6899507 30954305

[4] Ni Mhurchu C (2013). Monitoring the availability of healthy and unhealthy foods and non-alcoholic beverages in community and consumer retail food environments globally. Obes Rev.

[5] Anand SS (2015). Food consumption and its impact on cardiovascular disease: importance of solutions focused on the globalized food system: a report from the workshop convened by the World Heart Federation. J Am Coll Cardiol.

[6] Worldpanel K. Winning omnichannel: the future of FMCG and retail post-COVID. 2021, Kantar: London, UK.

[7] Office for National Statistics, *Retail Sales Index internet sales (20 August 2021)*. 2021, Office for National Statistics: Newport, Wales.

[8] Australian Associated Press, Lockdown boom in online grocery shopping, in Australian Associated Press. 2021.

[9] • Sacks G, Robinson E, Cameron AJ. Issues in measuring the healthiness of food environments and interpreting relationships with diet, obesity and related health outcomes. Curr Obes Rep. 2019;8(2):98–111. **This review article clearly highlights the need for comprehensive and standardised frameworks and methods to monitor food environments and better understand their impact on diets and health outcomes.** 10.1007/s13679-019-00342-4 30879246

[10] Jaenke R (2021). Development and pilot of a tool to measure the healthiness of the in-store food environment. Public Health Nutr.

[11] Lytle L, Myers A (2017). Measures registry user guide: food environment.

[12] Kelly B (2013). Monitoring food and non-alcoholic beverage promotions to children. Obes Rev.

[13] Lee A (2013). Monitoring the price and affordability of foods and diets globally. Obes Rev.

[14] Bhatnagar P (2021). Are food and drink available in online and physical supermarkets the same? A comparison of product availability, price, price promotions and nutritional information. Public Health Nutr.

[15] Cameron AJ (2017). Do the foods advertised in Australian supermarket catalogues reflect national dietary guidelines?. Health Promot Int.

[16] Furey S. et al. What’s on offer? The types of food and drink on price promotion in retail outlets in the Republic of Ireland. SafeFood: Cork, Ireland. 2019.

[17] Goulding T, Lindberg R, Russell CG. The affordability of a healthy and sustainable diet: an Australian case study. Nutr J. 2020;19(1). 10.1186/s12937-020-00606-z PMC7528590 32998734

[18] Harrington RA, et al. Nutrient composition databases in the age of big data: FoodDB, a comprehensive, real-time database infrastructure. BMJ Open. 2019. 10.1136/bmjopen-2018-026652 PMC6609072 31253615

[19] Hillen J. Psychological pricing in online food retail*.* Br Food J. 2021.

[20] • Obesity Action Scotland. Survey of food and drink promotions in an online retail environment.Obes Act Scotland. 2021. **This study of price and non-monetary promotions in online supermarkets in Scotland included some focus on unique aspects of the setting, such as placement of promotions and online store layout and navigation.**

[21] Ogundijo DA, Tas AA, Onarinde BA (2021). An assessment of nutrition information on front of pack labels and healthiness of foods in the United Kingdom retail market. BMC Public Health.

[22] • Olzenak K, et al. How online grocery stores support consumer nutrition information needs. J Nutr Educ Behav. 2020;52(10):952–7. **This study of the information made available in online supermarkets in the USA included a specific focus on placement of nutrition information by retailers and user ability to customise the environment or use nutrition attributes to identify products.** 10.1016/j.jneb.2020.07.009 PMC7538868 33039023

[23] Pearson AL, et al. Obtaining fruit and vegetables for the lowest prices: pricing survey of different outlets and geographical analysis of competition effects*.* Plos One. 2014;9(3). 10.1371/journal.pone.0089775 PMC3961225 24651475

[24] Pereira RC, de Angelis-Pereira MC, Carneiro JDS (2019). Exploring claims and marketing techniques in Brazilian food labels. Br Food J.

[25] Pereira RC, Souza Carneiro JD, de Angelis Pereira MC. Evaluating nutrition quality of packaged foods carrying claims and marketing techniques in Brazil using four nutrient profile models*.* J Food Sci Technol. 2021. 10.1007/s13197-021-05162-w PMC8882526 35250075

[26] Price RK, et al. What foods are Northern Ireland supermarkets promoting? A content analysis of supermarket online. 2017.

[27] Riesenberg D (2019). Price promotions by food category and product healthiness in an Australian supermarket chain, 2017–2018. Am J Public Health.

[28] Stones C (2016). Online food nutrition labelling in the UK: how consistent are supermarkets in their presentation of nutrition labels online?. Public Health Nutr.

[29] Zorbas C (2019). The frequency and magnitude of price-promoted beverages available for sale in Australian supermarkets. Aust N Z J Public Health.

[30] Zorbas C (2021). Streamlined data-gathering techniques to estimate the price and affordability of healthy and unhealthy diets under different pricing scenarios. Public Health Nutr.

[31] •• Khandpur, N., et al., Supermarkets in cyberspace: a conceptual framework to capture the influence of online food retail environments on consumer behavior. Int J Environ Res Public Health. 2020;17(22). **This is the first and, to date, only conceptual framework available which attempts to specifically and comprehensively capture those aspects of online retail food environments that influence consumer purchases.** 10.3390/ijerph17228639 PMC7699869 33233798

[32] Breugelmans E, Campo K (2011). Effectiveness of in-store displays in a virtual store environment. J Retail.

[33] Benn Y (2015). What information do consumers consider, and how do they look for it, when shopping for groceries online?. Appetite.

[34] Vadiveloo MK (2021). Contributions of food environments to dietary quality and cardiovascular disease risk. Curr Atheroscler Rep.

[35] Afshin A (2015). CVD prevention through policy: a review of mass media, food/menu labeling, taxation/subsidies, built environment, school procurement, worksite wellness, and marketing standards to improve diet. Curr Cardiol Rep.

[36] Gertner D (2016). Calories and cents:customer value and the fight against obesity. Soc Mark Q.

[37] Bennett R (2020). Prevalence of healthy and unhealthy food and beverage price promotions and their potential influence on shopper purchasing behaviour: a systematic review of the literature. Obes Rev.

[38] Croker H (2020). Front of pack nutritional labelling schemes: a systematic review and meta-analysis of recent evidence relating to objectively measured consumption and purchasing. J Hum Nutr Diet.

[39] El-Abbadi NH (2020). Nutrient profiling systems, front of pack labeling, and consumer behavior. Curr Atheroscler Rep.

[40] Jones A (2019). Front-of-pack nutrition labelling to promote healthier diets: current practice and opportunities to strengthen regulation worldwide. BMJ Glob Health.

[41] Kaur A (2016). The nutritional quality of foods carrying health-related claims in Germany, The Netherlands, Spain, Slovenia and the United Kingdom. Eur J Clin Nutr.

[42] Kaur A (2016). How many foods in the UK carry health and nutrition claims, and are they healthier than those that do not?. Public Health Nutr.

[43] Franco-Arellano B, et al. Examining the nutritional quality of Canadian packaged foods and beverages with and without nutrition claims. Nutrients. 2018;10(7). 10.3390/nu10070832 PMC6073495 29954102

[44] Pulker CE, Scott JA, Pollard CM (2018). Ultra-processed family foods in Australia: nutrition claims, health claims and marketing techniques. Public Health Nutr.

[45] Borges CA, Gabe KT, Jaime PC (2021). Consumer food environment healthiness score: development, validation, and testing between different types of food retailers. Int J Environ Res Public Health.

[46] Vandevijvere S (2018). Towards healthier supermarkets: a national study of in-store food availability, prominence and promotions in New Zealand. Eur J Clin Nutr.

[47] Harmer G, et al. Capturing the healthfulness of the in-store environments of United Kingdom supermarket stores over 5 months (January–May 2019). Am J Prevent Med. 2021. 10.1016/j.amepre.2021.04.012 34158196

[48] Sacks G, SS, Grigsby-Duffy L, Robinson E, Orellana L, Marshall J, Cameron AJ. Inside our supermarkets: assessment of the healthiness of Australian supermarkets, Australia 2020. Deakin University: Melbourne. 2020.

[49] Munson J, Tiropanis T, Lowe M. Online grocery shopping: identifying change in consumption practices. 2017.

[50] Wang RJ-H, Malthouse EC, Krishnamurthi L (2015). On the go: how mobile shopping affects customer purchase behavior. J Retail.

[51] Swinburn B (2013). INFORMAS (International Network for Food and Obesity/non-communicable diseases Research, Monitoring and Action Support): overview and key principles. Obes Rev.

