# Supplementary material for: Evidence Gaps in Assessments of the Healthiness of Online Supermarkets Highlight the Need for New Monitoring Tools: a Systematic Review
Source: Curr Atheroscler Rep. 2022 Feb 9;24(4):215–33. doi: 10.1007/s11883-022-01004-y (PMC9023389; doi:10.1007/s11883-022-01004-y)

# Supplementary material

**Supplementary Table 1. Search terms**

| Embase | |
| --- | --- |
| # | Syntax |
| 1 | (supermarket* or (food adj2 (retail* or vendor* or outlet* or sale* or sell* or market* or store* or shop* or outlet*)) or (grocer* adj2 (shop* or store*))).ab,kw,ti. |
| 2 | (digital or e-store* or webstore* or website* or web-based or online or ((online or internet or web) adj2 (shop* or store* or site*))).ab,kw,ti. |
| 3 | (((product* or food*) adj2 (availab* or access* or pric* or cost* or promot* or place* or market* or purchas* or health*)) or ((food or nutrition* or eating or diet*) adj2 environment*)).ab,kw,ti. |
| 4 | 1 and 2 and 3 |
| 5 | limit 4 to yr="2010 -Current" |
|  | |
| PubMed | |
| # | Syntax |
| 1 | "supermarket*"[Title/Abstract] OR "food retail*"[Title/Abstract] OR "food vendor*"[Title/Abstract] OR "food outlet*"[Title/Abstract] OR "food sale*"[Title/Abstract] OR "food sell*"[Title/Abstract] OR "food market*"[Title/Abstract] OR "food store*"[Title/Abstract] OR "food shop*"[Title/Abstract] OR "food outlet*"[Title/Abstract] OR ("grocer*"[Title/Abstract] AND "shop*"[Title/Abstract]) OR ("grocer*"[Title/Abstract] AND "store*"[Title/Abstract]) |
| 2 | "digital"[Title/Abstract] OR "e store*"[Title/Abstract] OR "webstore*"[Title/Abstract] OR "website*"[Title/Abstract] OR "web-based"[Title/Abstract] OR "online"[Title/Abstract] OR "online shop*"[Title/Abstract] OR "online store*"[Title/Abstract] OR "online site*"[Title/Abstract] OR "internet shop*"[Title/Abstract] OR "internet store*"[Title/Abstract] OR "internet site*"[Title/Abstract] OR "web shop*"[Title/Abstract] OR "web store*"[Title/Abstract] OR "web site*"[Title/Abstract] |
| 3 | ("product availab*"[Title/Abstract] OR "product access*"[Title/Abstract] OR "product pric*"[Title/Abstract] OR "product cost*"[Title/Abstract] OR "product promot*"[Title/Abstract] OR "product place*"[Title/Abstract] OR "product market*"[Title/Abstract] OR "product purchas*"[Title/Abstract] OR "product health*"[Title/Abstract] OR "food availab*"[Title/Abstract] OR "food access*"[Title/Abstract] OR "food pric*"[Title/Abstract] OR "food cost*"[Title/Abstract] OR "food promot*"[Title/Abstract] OR "food place*"[Title/Abstract] OR "food market*"[Title/Abstract] OR "food purchas*"[Title/Abstract] OR "food health*"[Title/Abstract]) or ("food environment*"[Title/Abstract] OR "nutrition environment*"[Title/Abstract] OR "eating environment*"[Title/Abstract] OR "diet environment*"[Title/Abstract]) |
| 4 | ("supermarket*"[Title/Abstract] OR "food retail*"[Title/Abstract] OR "food vendor*"[Title/Abstract] OR "food outlet*"[Title/Abstract] OR "food sale*"[Title/Abstract] OR "food sell*"[Title/Abstract] OR "food market*"[Title/Abstract] OR "food store*"[Title/Abstract] OR "food shop*"[Title/Abstract] OR "food outlet*"[Title/Abstract] OR ("grocer*"[Title/Abstract] AND "shop*"[Title/Abstract]) OR ("grocer*"[Title/Abstract] AND "store*"[Title/Abstract])) and ("digital"[Title/Abstract] OR "e store*"[Title/Abstract] OR "webstore*"[Title/Abstract] OR "website*"[Title/Abstract] OR "web-based"[Title/Abstract] OR "online"[Title/Abstract] OR "online shop*"[Title/Abstract] OR "online store*"[Title/Abstract] OR "online site*"[Title/Abstract] OR "internet shop*"[Title/Abstract] OR "internet store*"[Title/Abstract] OR "internet site*"[Title/Abstract] OR "web shop*"[Title/Abstract] OR "web store*"[Title/Abstract] OR "web site*"[Title/Abstract]) and (("product availab*"[Title/Abstract] OR "product access*"[Title/Abstract] OR "product pric*"[Title/Abstract] OR "product cost*"[Title/Abstract] OR "product promot*"[Title/Abstract] OR "product place*"[Title/Abstract] OR "product market*"[Title/Abstract] OR "product purchas*"[Title/Abstract] OR "product health*"[Title/Abstract] OR "food availab*"[Title/Abstract] OR "food access*"[Title/Abstract] OR "food pric*"[Title/Abstract] OR "food cost*"[Title/Abstract] OR "food promot*"[Title/Abstract] OR "food place*"[Title/Abstract] OR "food market*"[Title/Abstract] OR "food purchas*"[Title/Abstract] OR "food health*"[Title/Abstract]) or ("food environment*"[Title/Abstract] OR "nutrition environment*"[Title/Abstract] OR "eating environment*"[Title/Abstract] OR "diet environment*"[Title/Abstract])) |
| 5 | Filters: from 2010 - 2021 |
|  | |
| Cinahl | |
| # | Syntax |
| 1 | TI (supermarket* or (food N2 (retail* or vendor* or outlet* or sale* or sell* or market* or store* or shop* or outlet*)) or (grocer* N2 (shop* or store*))) OR AB (supermarket* or (food N2 (retail* or vendor* or outlet* or sale* or sell* or market* or store* or shop* or outlet*)) or (grocer* N2 (shop* or store*))) |
| 2 | TI (digital or e-store* or webstore* or website* or web-based or online or ((online or internet or web) N2 (shop* or store* or site*))) OR AB (digital or e-store* or webstore* or website* or web-based or online or ((online or internet or web) N2 (shop* or store* or site*))) |
| 3 | TI ((product* or food*) N2 (availab* or access* or pric* or cost* or promot* or place* or market* or purchas* or health*)) OR AB ((product* or food*) N2 (availab* or access* or pric* or cost* or promot* or place* or market* or purchas* or health*)) |
| 4 | TI ((food or nutrition* or eating or diet*) N2 environment*) OR AB ((food or nutrition* or eating or diet*) N2 environment*) |
| 5 | S3 or S4 |
| 6 | S1 and S2 and S5 |
| 6 | Date Published: 20100101-20211231 |
|  | |
| Cochrane Library | |
| # | Syntax |
| 1 | (supermarket* or (food adj (retail* or vendor* or outlet* or sale* or sell* or market* or store* or shop* or outlet*)) or (grocer* adj (shop* or store*))):ab,kw,ti |
| 2 | (digital or e-store* or webstore* or website* or web-based or online or ((online or internet or web) adj (shop* or store* or site*))):ab,kw,ti |
| 3 | (((product* or food*) adj (availab* or access* or pric* or cost* or promot* or place* or market* or purchas* or health*)) or ((food or nutrition* or eating or diet*) adj environment*)):ab,kw,ti |
| 4 | #1 and #2 and #3 |
|  | |
| Scopus | |
| # | Syntax |
| 1 | TITLE-ABS-KEY ( supermarket* OR ( food W/2 ( retail* OR vendor* OR outlet* OR sale* OR sell* OR market* OR store* OR shop* OR outlet* ) ) OR ( grocer* W/2 ( shop* OR store* ) ) ) |
| 2 | TITLE-ABS-KEY ( digital OR e-store* OR webstore* OR website* OR web-based OR online OR ( ( online OR internet OR web ) W/2 ( shop* OR store* OR site* ) ) ) |
| 3 | TITLE-ABS-KEY ( ( ( food OR nutrition* OR eating OR diet* ) W/2 environment* ) OR ( ( ( product* OR food* ) W/2 ( availab* OR access* OR pric* OR cost* OR promot* OR place* OR market* OR purchas* OR health* ) ) ) ) |
| 4 | TITLE-ABS-KEY (( supermarket* OR ( food W/2 ( retail* OR vendor* OR outlet* OR sale* OR sell* OR market* OR store* OR shop* OR outlet* ) ) OR ( grocer* W/2 ( shop* OR store* ) ) ) and ( digital OR e-store* OR webstore* OR website* OR web-based OR online OR ( ( online OR internet OR web ) W/2 ( shop* OR store* OR site* ) ) ) and ( ( ( food OR nutrition* OR eating OR diet* ) W/2 environment* ) OR ( ( ( product* OR food* ) W/2 ( availab* OR access* OR pric* OR cost* OR promot* OR place* OR market* OR purchas* OR health* ) ) ) )) |
| 5 | PUBYEAR > 2009 |
|  | |
| Web of Science | |
| 1 | TI=(supermarket* or (food NEAR/2 (retail* or vendor* or outlet* or sale* or sell* or market* or store* or shop* or outlet*) ) or (grocer* NEAR/2 (shop* or store*) )) |
| 2 | AB=(supermarket* or (food NEAR/2 (retail* or vendor* or outlet* or sale* or sell* or market* or store* or shop* or outlet*) ) or (grocer* NEAR/2 (shop* or store*) )) |
| 3 | #2 OR #1 |
| 4 | TI=(digital or e-store* or webstore* or website* or web-based or online or ((online or internet or web) NEAR/2 (shop* or store* or site*) )) |
| 5 | AB=(digital or e-store* or webstore* or website* or web-based or online or ((online or internet or web) NEAR/2 (shop* or store* or site*) )) |
| 6 | #4 OR #5 |
| 7 | TI=((product* or food*) NEAR/2 (availab* or access* or pric* or cost* or promot* or place* or market* or purchas* or health*) ) |
| 8 | AB=((product* or food*) NEAR/2 (availab* or access* or pric* or cost* or promot* or place* or market* or purchas* or health*) ) |
| 9 | TI=((food or nutrition* or eating or diet*) NEAR/2 environment*) |
| 10 | AB=((food or nutrition* or eating or diet*) NEAR/2 environment*) |
| 11 | #7 OR #8 OR #9 OR #10 |
| 12 | #3 AND #6 AND #11 |
| 13 | 2010 on |

**Supplementary Figure 1. Study search and inclusion**


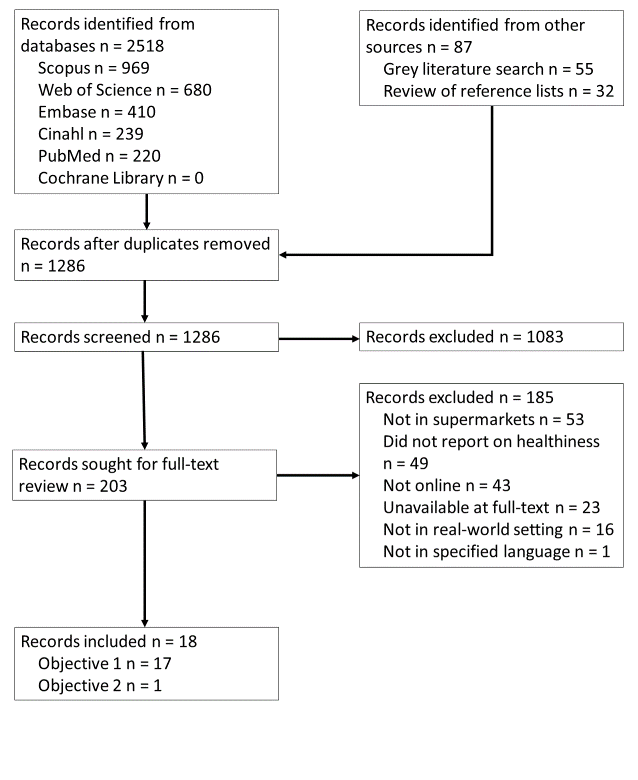

Supplement: Supplementary file 1 — Supplementary file1 (DOCX 50 kb) [file 11883_2022_1004_MOESM1_ESM.docx]
